# Inactivating SARS-CoV-2 Using 275 nm UV-C LEDs through a Spherical Irradiation Box: Design, Characterization and Validation

**DOI:** 10.3390/ma14092315

**Published:** 2021-04-29

**Authors:** Nicola Trivellin, Matteo Buffolo, Francesco Onelia, Alberto Pizzolato, Marco Barbato, Viviana Teresa Orlandi, Claudia Del Vecchio, Fabrizio Dughiero, Enrico Zanoni, Gaudenzio Meneghesso, Andrea Crisanti, Matteo Meneghini

**Affiliations:** 1 Department of Industrial Engineering, University of Padova, Via Gradenigo 6A, 35131 Padova, Italy; fabrizio.dughiero@unipd.it; 2 Department of Information Engineering, University of Padova, Via Gradenigo 6B, 35131 Padova, Italy; matteo.buffolo@unipd.it (M.B.); marco.barbato@light-cube.com (M.B.); enrico.zanoni@unipd.it (E.Z.); gaudenzio.meneghesso@unipd.it (G.M.); matteo.meneghini@unipd.it (M.M.); 3 Department of Molecular Medicine, University of Padova, Via Gabelli 63, 35121 Padova, Italy; francesco.onelia@studenti.unipd.it (F.O.); claudia.delvecchio@unipd.it (C.D.V.); andrea.crisanti@unipd.it (A.C.); 4 LightCube SRL, Viale Navigazione Interna 51, 35129, Padova, Italy; alberto.pizzolato@light-cube.com; 5 DBSV, University of Insubria, Via J.H. Dunant 3, 21100 Varese, Italy; viviana.orlandi@uninsubria.it

**Keywords:** SARS-CoV-2, COVID-19, LED, UVC, disinfection, inactivation, virucide

## Abstract

We report on the design, characterization and validation of a spherical irradiation system for inactivating SARS-CoV-2, based on UV-C 275 nm LEDs. The system is designed to maximize irradiation intensity and uniformity and can be used for irradiating a volume of 18 L. To this aim: (i) several commercially available LEDs have been acquired and analyzed; (ii) a complete optical study has been carried out in order to optimize the efficacy of the system; (iii) the resulting prototype has been characterized optically and tested for the inactivation of SARS-CoV-2 for different exposure times, doses and surface types; (iv) the result achieved and the efficacy of the prototype have been compared with similar devices based on different technologies. Results indicate that a 99.9% inactivation can be reached after 1 min of treatment with a dose of 83.1 J/m^2^.

## 1. Introduction

The spread of SARS-CoV-2 virus, the etiological agent of COVID-19, is stressing the global healthcare [1] and economic systems [2]. Respiratory viruses can be transmitted by droplets and aerosol that can be generated just by normal speaking [3]. While personal protection devices such as face masks are effective toward the spread of droplets, for example, a fluid-resistant (Type-IIR) surgical facemask is expected to reduce the droplet dispersion by at least 80% [4,5,6], contamination of surfaces [7], water and air must also be prevented to minimize the spread of the virus. In most cases, decontamination is performed through chemical agents, like alcohol- or chlorine-based solutions. However, these disinfection strategies are time-consuming, and not suitable for large surfaces. 

For this reason, non-chemical and photo-assisted strategies are currently being explored, mostly based on ultraviolet light. Several papers indicated that UV light can be effective towards the inactivation of different pathogenic microorganisms, including, recently, the SARS-CoV-2 [8] on surfaces. UVC irradiation allows a precise and reliable sanitization of several viruses and bacteria [9], thus helping the use of “community” objects like portable payment terminals, handrails, elevators, etc.

UV-assisted disinfection is of particular importance in community environments, especially in the presence of young generations; schools, gyms and sport fields are often kept open to relieve social tension, thus being places where the virus can easily spread. In addition, team sport activities are affected by the necessary use of “community” objects like sport balls, that are touched by all players, in the presence of droplets, aerosol and body fluids. For this reason, providing frequently sanitized sport equipment (like basketballs) is crucial for the containment of the contagion and the continuation of the physical activities. Most personal objects used at school have dimensions of 100–200 mm, while sport-balls can have a diameter near 250 mm, so designing systems capable of inactivating the viruses in volumes around 15–18 L is a necessity to control the virus in community life.

Deep ultraviolet LEDs based on aluminum gallium nitride (AlGaN) material have been available for approximately two decades [10]. Nevertheless, their limited performances [11], reliability [12] and high cost per watt played a major role in limiting their market diffusion. Recently, the efficiency of these devices has risen up to levels of 1–3%, while a sensible reduction of cost (in the order of 300–400 € per optical watt at the time of writing) made them suitable for different commercial applications. UVC LEDs are still less efficient than low pressure mercury lamp, but their compactness, low voltage operation and instant switch on compensate for these deficiencies.

The goal of this paper is to present the design, optimization and validation of a sanitization system based on UVC-LEDs, specifically tested with reference to the SARS-CoV-2 virus. The proposed system is designed to inactivate the virus located on the surface of personal and community objects, in particular spherical sport balls, thus being ideal for the disinfection in community environments (sport fields, schools, etc.).

## 2. Experimental Details

The light-emitting part of the system is represented by commercially available off-the-shelf UVC LEDs. For the proposed study, we evaluated 6 UVC LEDs from different manufacturers, the details of the LEDs, as determined from each datasheet, are reported in Table 1.

UVC LED technology is still under development. From the technical and cost/manufacturing aspects, it becomes crucial to carefully estimate the electrical and optical properties of the available LEDs as a starting point for the design optimization.

In this work, we performed: (i) electrical characterization by means of current-voltage characteristics, (ii) optical characterization by means of absolute spectral measurement; (iii) efficiency analysis at different driving currents.

The electrical characterization was carried out by means of a Keithley 2614B Source-meter analyzer (Cleveland, OH, USA). For each LED, the voltage was swept between −2 V and the maximum rated voltage. The optical characterization was carried out by means of an absolute irradiance calibrated Ocean Insight USB4000 spectrometer (Orlando, FL, USA) equipped with a CC3-DA cosine corrector, and the spectral range is between 200 and 850 nm. In order to perform the optical flux measurement, the LEDs were mounted on a 2 axis stepper motor goniometer and rotated in order to calculate the radiated flux from irradiance measurements. To acquire a sufficient signal from the LEDs, the aperture of the spectrometer was positioned at a distance of 300 mm from the emitter. The power emitted from the LED was also measured at different current levels, in order to extract the efficiency of the different devices as a function of driving current.

The design of the irradiation chambers was carried out by means of ray-tracing simulations. For the implementation of the sources, a ray file has been calculated from the angular emission of the LEDs as previously measured. The optical simulations are aimed at calculating the spatial resolved irradiance on a sensor surface. The sensor surface is shaped as the target object: a sphere, representing the largest object to be irradiated (sportsball), other shapes (cube and flat surface) have also been evaluated.

The system proposed in this work is based on an openable spherical chamber, several UVC LEDs are positioned in the surface of the sphere with an inward emission. The material of the chamber is steel with the internal surface plated with chromium, the relative surface reflectivity used in the simulation has been calculated from the work of Sarosi et al. [13]; we estimated a reflectivity of 60% at 275 nm. Distributing uniform points on the surface of a sphere is a well-known problem [14] with no perfect solution.

**Figure 1:**
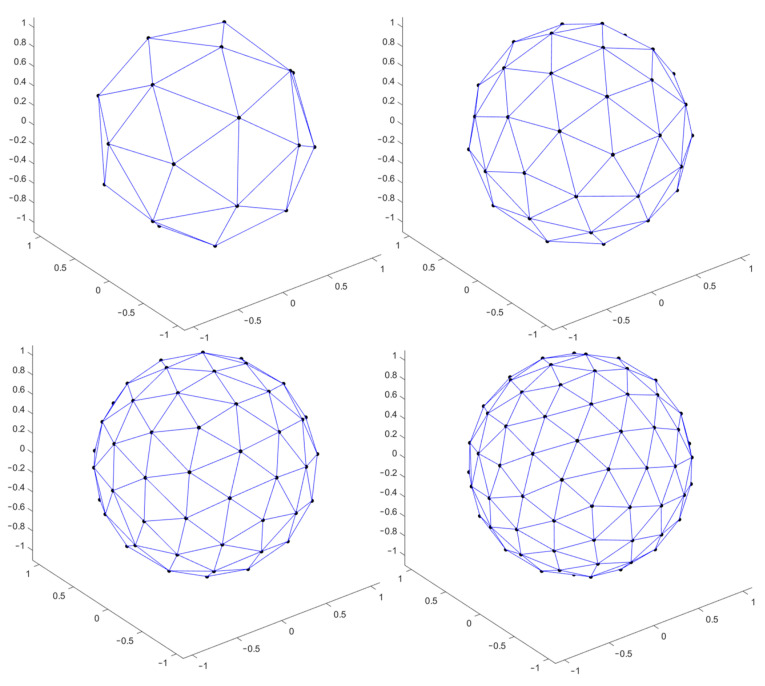
Calculated point distribution on the surface of a sphere (**top left**) 25 points, (**top right**) 50 points, (**bottom left**) 75 points, (**bottom right**) 100 points.

The Matlab (MathWorks, Natick, MA, USA) function ParticleSampleSphere [15] has been used to generate an approximately uniform triangular tessellation of a unit sphere (used at the beginning for minimizing the generalized electrostatic potential energy, i.e., Reisz s-energy, of a system of charged particles). Initial configuration of particles is based on random sampling of a sphere. Effectively, this function uses gradient descent and produces a locally optimal solution to the problem that involves finding a minimum Reisz s-energy configuration of N equally charged particles confined to the surface of a unit sphere. Figure 1 reports the point distributions as calculated by the Matlab function and used in the following of this work for the design of a spherical emitting surface.

## 3. Design Target

When the wavelength is fixed, the antiviral effectiveness of a UV system is related to a specific irradiation fluence, corresponding to a specific light energy per unit area. The final design target is to irradiate the entire surface of the object with a minimum fluence. Considering that, for the type of the systems analyzed in this work, the irradiation time is the same for the entire target surface, a minimum fluence value translates into a minimum irradiance value.

The target of the design is an optimization of the minimum irradiance on the target surface versus the cost of the system. For this analysis, the cost of the system is represented mainly by the cost of the single LED multiplied by the number of the LEDs in the system. On the market, LEDs emitting at a peak wavelength of 275 ± 10 nm are available with different radiant fluxes, typical values going from 10 mW for a 100 mA device to 110 mW for an 800 mA device. Considering that the price of these LEDs ($Price_{\mathrm{LED}}$) increases with their emitted optical power ($OP_{\mathrm{LED}}$) with a nearly linear dependence (defined $K_{p}$ factor in the following), we estimate that the price of the system ($Price_{\mathrm{sys}}$) composed of *N* LEDs is related to the total optical power ($OP_{\mathrm{sys}}$) emitted and that the LEDs all have the same power.

(1) $$\begin{array}{c} OP_{\mathrm{sys}}=N\times OP_{\mathrm{LED}} \end{array}$$

(2) $$\begin{array}{c} Price_{\mathrm{LED}}=K_{p}\times OP_{\mathrm{LED}} \end{array}$$

(3) $$\begin{array}{c} Price_{\mathrm{sys}}=N\times Price_{\mathrm{LED}}=N\times K_{p}\times OP_{\mathrm{LED}}=K_{P}\times OP_{\mathrm{sys}} \end{array}$$

For a finer assessment of the costs, it should be noted that by increasing the number of LEDs, manufacturing and assembly costs also increase; we will therefore carefully optimize the LED number.

For a fixed treatment time, the design task translates into the maximization of the minimum irradiance on the target surface for a constant total installed optical power. Since a local irradiance higher than the minimum is not necessary, the immediate annotation is that to improve the minimum irradiance, we should also improve the irradiance uniformity, in order not to waste any optical power.

### Optical Design

From the recent scientific literature, the amount of fluence necessary to inactivate the Sars-CoV-2 is estimated at different values approximately between 6–8 mJ/cm^2^ [16], up to 37.5 mJ/cm^2^ [17]. Our aim is to inactivate the virus in a few minutes, possibly just 60 s. We set the ideal average irradiance at 2.5 W/m^2^ and a target minimum irradiance of at least 1 W/m^2^. The ideal average irradiance does not consider the actual optical losses, while the minimum irradiance is the target value for the optimization we will use in the following.

In the design of the system, several possible architectures have been evaluated, but for manufacturing simplicity a design constraint has been set. LEDs are positioned onto a spherical surface with a diameter of 330 mm, the optical axis of each LED is pointing at the center of the sphere. We will optimize the number of the LEDs together with their optical power. The uniformity will be evaluated for 25, 50, 75 and 100 LEDs, while the sum of the power emitted by the LEDs will be approximately 500 mW. We will also investigate the effect of the diameter of the internal sphere with respect to irradiance values. The best optimization solution will be the configuration allowing the higher minimum value of irradiance.

To analyze the data, we will use, among others, the non-uniformity NU parameter, defined as reported below:

(4) $$\begin{array}{c} NU=\frac{E_{\max}-E_{\min}}{E_{\max}+E_{\min}}\times 100\;\left(\%\right) \end{array}$$

## 4. Biological Materials and Assays

### 4.1. Viral Stock Preparation and Viral Titer Evaluation

The photo-inactivation tests were performed against virus belonging to the family of *Coronaviridae*, SARS-CoV-2. All the experiments were performed in a biosafety level 3 laboratory, while the virus was obtained from ISS (Istituto Superiore della Sanità, Roma, Italy). Each viral suspension was prepared and propagated on a large scale in monolayer cell cultures of Vero E6 ATCC CCL-81™ (American Type Culture Collections). Cell cultures were incubated at 37 °C with 5% (*v*/*v*) CO_2_ in DMEM (Dulbecco’s Modified Eagle Medium) added with 10% (*v*/*v*) of fetal bovine serum (FBS), 1% (*w*/*v*) of penicillin-streptomycin.

Viral titer was calculated using the Spaerman-Kärber equation (ID_50_ assessment), as follows:

(5) $$\begin{array}{c} -\log_{10}{\mathrm{ID}}_{50}=-\left(x_{0}\right)-\left\{\left[\frac{R}{100}\right]-\;0.5\right\}\times -\log_{10}\;of\;the\;dilution\;factor \end{array}$$

where $x_{0}$ is the 10th base logarithm of the lowest dilution with 100% positive reaction and cytopathic effect (CPE) R is the summation (%) of positive cultures.

### 4.2. Photo-Virucidal Test

The photo-virucidal test was performed at 20° C. A volume of 50 µL of the viral suspension (10^7^ PFU/mL, Plaque Forming Units per milliliter) has been inoculated directly on three different samples: a basketball, a soccer ball and a volleyball. The suspension was allowed to dry, and then the sample has been placed inside the irradiation chamber. A 35 mm stainless steel support disc and the three balls were inoculated with a viral suspension (50 µL) and not irradiated and used as control. At the moment of the publication of this work, no standardized regulations have been identified for testing of Sars-CoV-2 virucidal efficacy, the stainless-steel disc has then been used to comply with the scientific methodologies used in the virological field [18], while to correctly estimate the virucidal effect of the prototype, the un-irradiated sport ball has been used as control.

After UVC irradiation (1 min and 2 min), a sterile swab was used to recover the residual virus from the sample surface and the surface of the control. Thereafter, the swab was inoculated in 1 mL of fresh medium and ten-fold dilutions (from 10^−2^ to 10^−9^) were prepared. Each dilution was used to inoculate six wells of 24-well plates containing confluent VERO culture (>90%). Six wells of the plate were untreated and considered as controls. Cells were incubated at 37 °C for 1 h, and subsequently the supernatant culture was removed. Cells were washed with PBS and added with fresh medium (500 µL of DMEM supplemented with 2% *v/v* FBS and 0.75% *v/v* of carboxymethylcellulose).

After incubation at 37 °C for three days, the plaque formation was checked, and upon Crystal Violet staining, the infectivity title (ID_50_) was determined with the Spaerman-Kärber equation previously shown (5). A decrease of ≥4 log unit of viral titer (pfu/mL) can be considered a virucidal effect [19].

In order to verify the reproducibility, the virucidal tests have been repeated three times for each of the three balls, and for each test duration, 18 times in total, the reported values indicate the average of the three tests for each condition.

## 5. Results

LED Characterization Results

**Figure 2:**
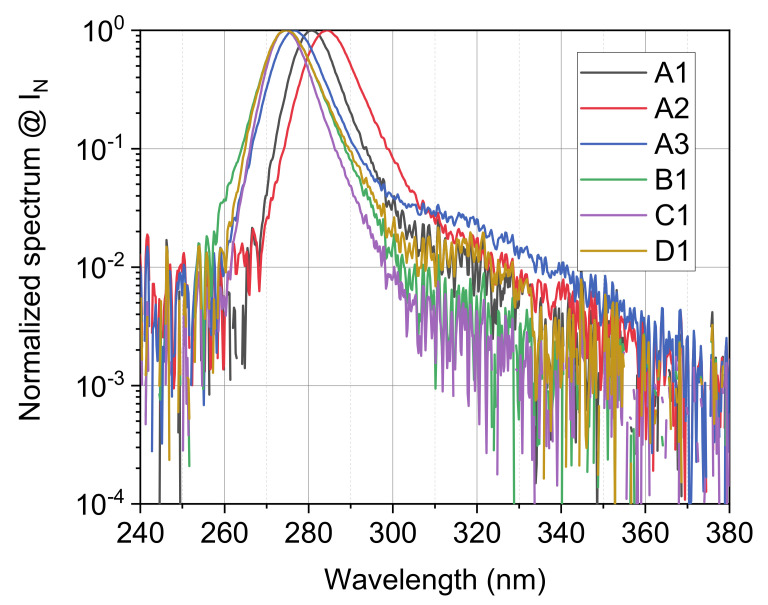
Normalized spectrum of the selected LEDs, semi logarithmic scale, LEDs measured at nominal current, 25 °C.

**Figure 3:**
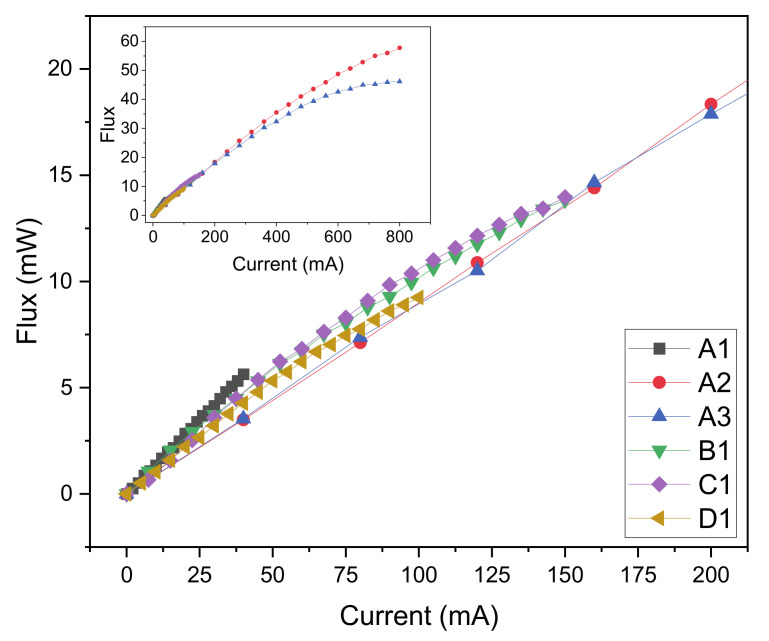
Optical flux as a function of current for the selected LEDs.

The electroluminescence spectrums of the analyzed LEDs are reported in Figure 2, they have been measured at the nominal current of each LED, normalized to the maximum value and plotted in a semi-log scale. Results indicate that the peak emission wavelength ranges from 275 nm for LEDs B1, C1, D1 and 284 nm for LED A2. LED A3 shows an evident higher wavelength emission, thus indicating a possible higher defect density related to Mg acceptors and nitrogen vacancies [20]. Results of the LED characterized optical power are reported in Figure 3. While LED A2 reports the higher optical power, we focus on the optical power range around 10 mW to evaluate an LED candidate for the next phase; in this range B1 and C1 report the higher value for the same current, with a slight advantage for C1.

**Figure 4:**
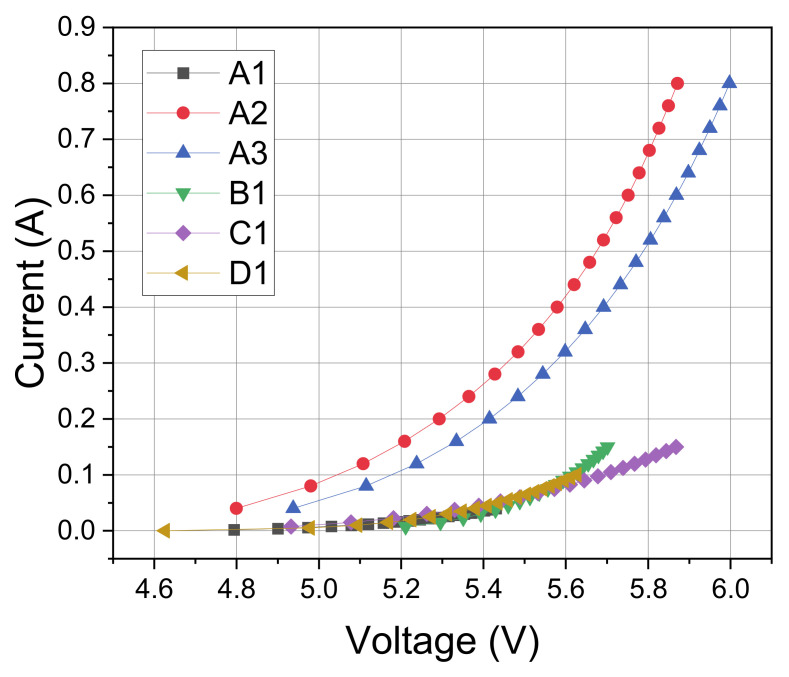
Electrical characteristics of the selected LEDs.

**Figure 5:**
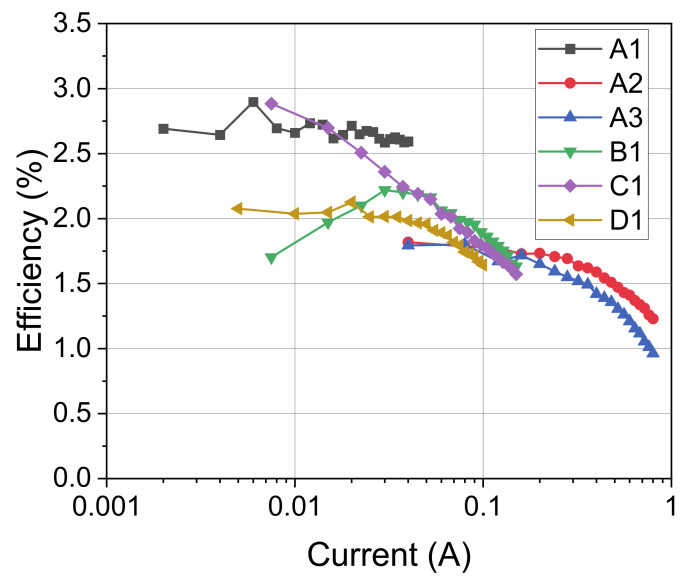
Wall plug efficiency as a function of LED current for the selected samples.

The results of the electrical characterization are reported in Figure 4, due to their larger chip size, LEDs A2 and A3 show a much lower voltage for the same current. For the other LEDs, results are similar up to 100 mA, with a V_f_ (forward voltage of the diode) of 5.6 V. Above this current, the higher series resistance of LED C1 has an important effect on voltage drop with respect to D1. As reported in the efficiency plot (Figure 5), the analyzed devices show an efficiency between 1% and 3%. Interestingly, LEDs with smaller chip (LED A1) have higher efficiency with respect to bigger LEDS (A2 and A3), the analyzed LEDs have similar efficacy at around 100 mA with values of approximately 1.75%. All the analyzed LEDs suffer from efficiency droop [21], but the amount of the droop is strongly dependent on the LED series resistance, for example, as seen between B1 and C1. It is also possible to notice that LED B1 has a lower efficiency at lower currents, possibly indicating that for this device, a higher defect density increases the non-radiative recombination rate, thus limiting the efficiency at low injection levels [21].

## 6. Design

### 6.1. LED Choice

Based on the previous characterization phase, both LEDs B1 and C1 are good candidates for the design of the system. They have lower emission wavelength, higher OP at around 100 mA with respect to the other candidates. While B1 has higher efficiency and lower voltage, C1 has slightly higher OP. Due to commercial availability, LED C1 has been chosen for the development of the irradiation system.

### 6.2. Optical Design

**Figure 6:**
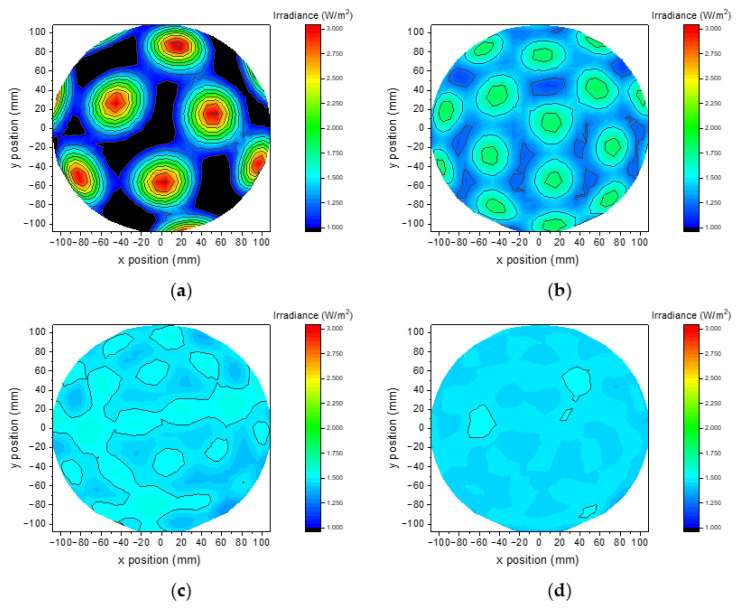
Optical simulation results: irradiance on the surface of the sphere, total optical power is constant at 500 mW, (**a**) 25 points, (**b**) 50 points, (**c**) 75 points, (**d**) 100 points.

**Figure 7:**
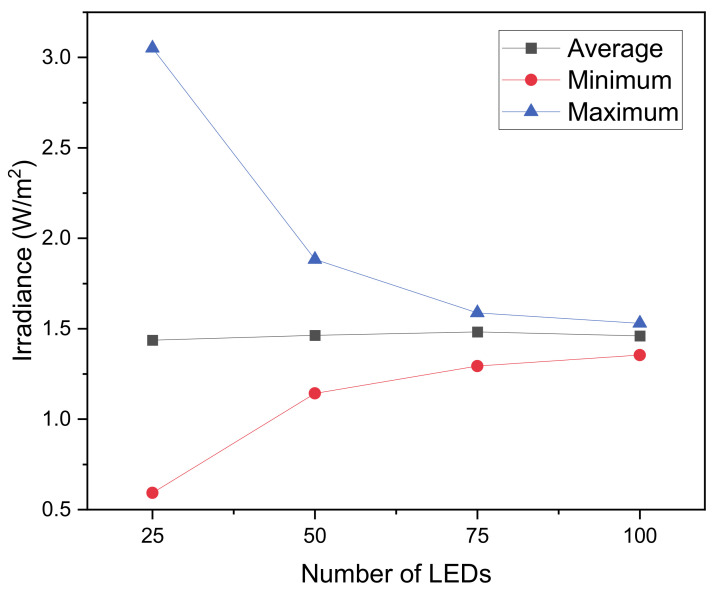
Simulated maximum, minimum and average irradiance as a function of the number of LEDs.

The results of the ray-tracing simulations are plotted in Figure 6; it can be observed that as the number of LEDs increases, the irradiance uniformity on the target surface also increases. The average irradiance is almost not affected due to the total optical power of the system being fixed at 500 mW in all the simulations. For a quantitative analysis, we report in Figure 7 the minimum, average and maximum for the 4 combinations. A massive variation in uniformity is detected between 25 and 50 LEDs, while increasing the number of sources above 50 provides a measurable, but minor improvement.

### 6.3. Selected Optical Design

Based on the simulation results, 56 LEDs have been selected as a design choice, just above the number of 50, as described in the previous section. To allow the subdivision of the sphere in two identical hemispheres with a 330 mm diameter, it has been necessary to slightly modify the position of the LEDs, the consequent reduction in the optimized uniformity has been compensated by the introduction of 6 more LEDs, bringing the total to 56. The optical power of the single LEDs has been kept at a nominal value of 10 mW, thus allowing a small increase in average irradiance.

**Figure 8:**
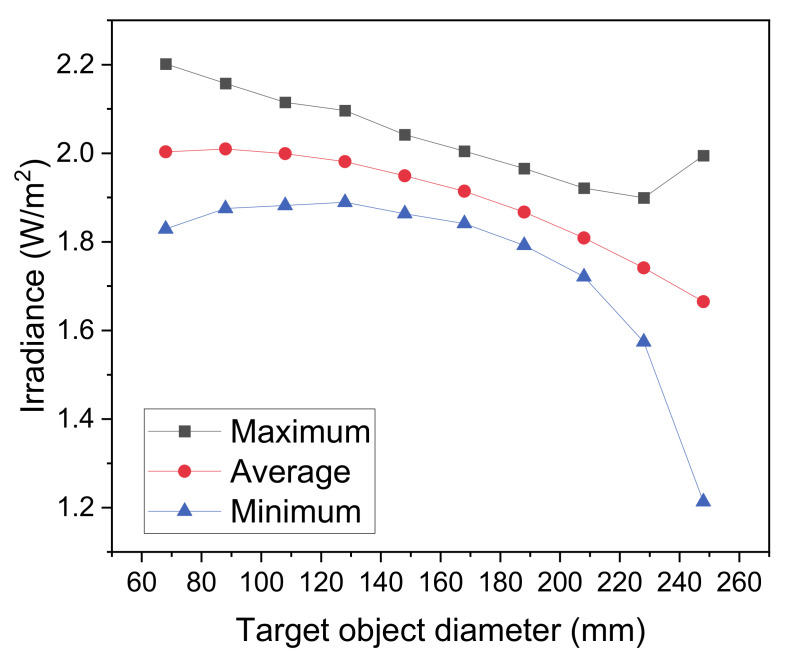
Irradiance as a function of the internal object diameter.

For the manufactured prototype, it has also been evaluated (Figure 8) the effect of the size of the irradiated sphere, we simulated the irradiance for spheres ranging from 65 mm (the size of a tennis ball) to 250 mm (the size of a basketball) placed at the center of the irradiation chamber. The concentric structure of the system implies that the reduction of the size of the target sphere allows an increase in the average irradiance, as well as a reduction of the NU on the surface of the target. The best condition is achieved for a target diameter sphere of 125 mm.

**Figure 9:**
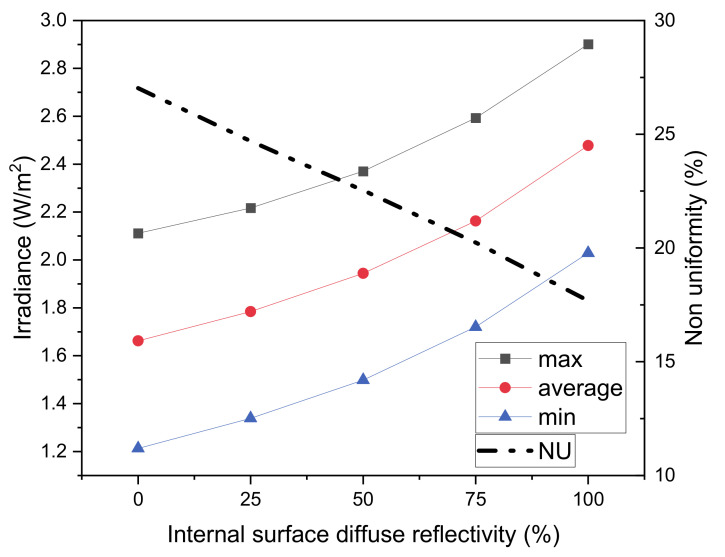
Irradiance and non-uniformity parameter (NU) as a function of internal surface reflectivity.

The manufacturing of the prototype has been carried out by means of two-chrome plated identical steel hemispheres. Since a spherical cavity with specular reflectivity would have the effect of guiding the rays, which are not incident onto the target surface in a circular loop, a diffused reflectivity has been chosen for the plating process. The effect of diffused reflectivity on the performance of the system has been reported in Figure 9. It can be noticed that as the reflectivity increases, the average irradiance increases in a more than linear fashion, the irradiance non uniformity of the system therefore reduces as reflectivity increases. With a chromium reflectivity of approximately 50% in the spectral region of interest, a non-uniformity of 23% is expected.

The results presented in Figure 8 also indicate that, due to the concentric irradiation of the chamber, the intensity is approximately constant in all the positions inside the sphere if the surface is outward directed. To analyze the effect of the shape of different objects to be irradiated, optical simulations have been carried out with two different targets: (i) a 176 mm × 176 mm flat square surface and (ii) a cube with a side of 144 mm, so that they are both inscribed in a sphere with a diameter of 250 mm. Both objects are positioned with a flat surface parallel to the ground and centered inside the disinfection chamber, they are simulated as 100% optically absorbent.

**Figure 10:**
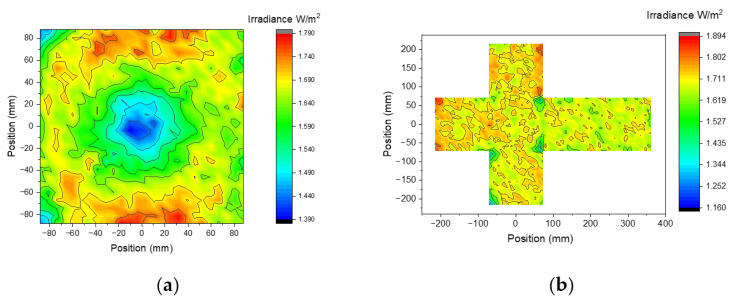
Simulated irradiance generated by the 56 LEDs prototype on a 176 × 176 cm^2^ flat surface (**a**) and on a cube with a side of 144 mm (net of the cube on the (**b**)), false color plot.

Results reported in Figure 10 indicate a good uniformity of the radiation, the minimum for the irradiance corresponds both to the vertex and the center of the flat surface, and have a substantially identical value with the one simulated on the spherical surface with a diameter of 250 mm. It can reasonably be concluded that the shape of the object does not influence the irradiance, as long as it is convex and centered with respect to the chamber.

### 6.4. Electronic Design, Safety and Robustness

The prototype is driven by standard constant current LED drivers with a high output voltage (up to 200 VDC) allowing the connection of several UVC LEDs in series. The system is regulated and managed by a microcontroller to automate the irradiation process; after the defined irradiation time has elapsed, the LEDs are switched off and the treatment is complete. Since short-wavelength light is dangerous both for the eyes and skin, when dealing with UVC LEDs, maximum care should be taken to avoid accidental human exposure. Both systems are equipped with safety mechanical switches to interrupt the LED circuit and to give feedback to the microcontroller. When the door is opened, the treatment may or may not be completed, the microcontroller resets the timer, and when the door is closed it starts over to ensure the designed dose is reached. The system has also been designed to ensure that in case of an LED failure (open circuit or short circuit), the microcontroller detects the voltage variation on the LED chain and reports a failure. The system is also equipped with a fan which circulates in the cavity between the external structure of the system and the internal surface where LEDs are positioned, lowering the LED temperature is crucial to improve efficiency and extend their lifetime [22].

## 7. Optical Characterization Results

**Figure 11:**
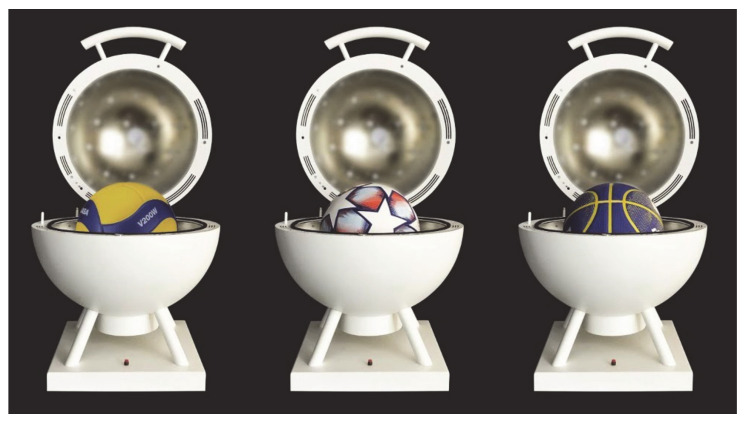
Picture of the manufactured prototype with the different tested balls.

**Figure 12:**
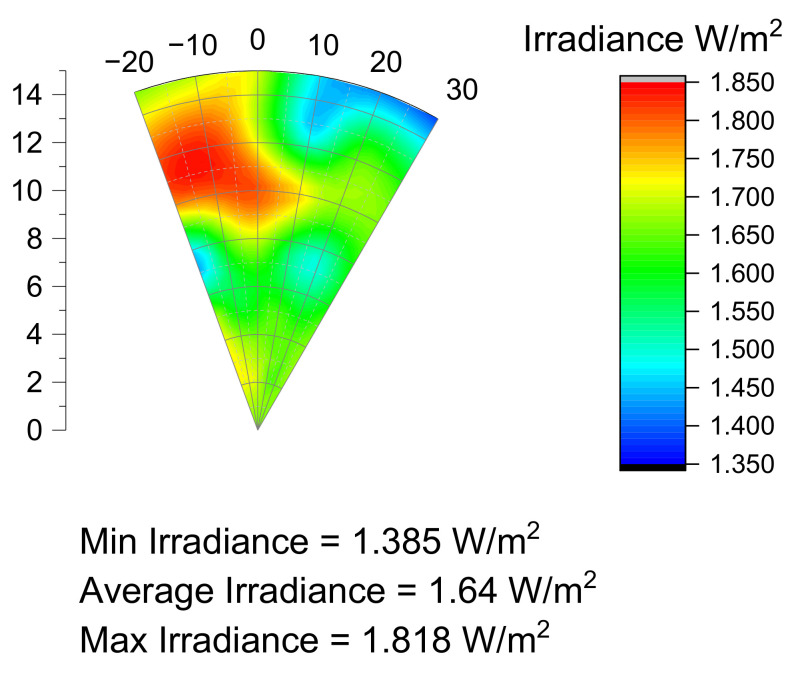
Measured uniformity on the surface of a 250 mm sphere wedge.

The characterization of the prototype reported in Figure 11 has been carried out by a spherical structure with a diameter of 250 mm, the USB4000 spectrometer is encased so that the cosine corrector is tangent to the spherical supporting structure. By rotating the spectrometer support, it has been possible to measure the spatial irradiance of a portion of the target surface. Results are presented in Figure 12. The measured minimum irradiance is similar to the simulated value, but since the maximum and average values are slightly lower, the NU is down to 13.5% in the sampled area.

## 8. Virucidal Tests Results

The virucidal test results are reported in Table 2. Three balls for soccer, basketball and volleyball were tested. Results indicate that for both 1 min and 2 min exposure time, the percent reduction after test contact time is greater than 99.9%, corresponding to more than log 3 inactivation. Percent reduction is calculated with respect to the titer of the corresponding non-irradiated control surface after the same duration. The reproducibility of the tests is verified by the 3 repetitions, each repetition has an after treatment viral titer of 0.01.

## 9. Considerations Related to the Prototype Usage and Running Costs

Although the major purpose of this work is the dissemination of the design procedure and the antiviral results achieved with respect to the sample fluence, it is also relevant to comment on the efficacy of the manufactured device and its running costs compared to other systems able to sanitize similar objects.

The proposed system is mainly intended to be used by non-specialized personnel of gyms and training centers to sanitize sport balls before and during both training and competition activities. Due to the COVID-19 pandemic and the related market request for disinfection apparatus, several other systems have been proposed for sport activities, some of them are based on adaptation of a general-purpose system, while others are specifically designed for sport balls; to our knowledge, they are all based on liquid disinfectants to be sprayed on the surface of the treated objects. In the following, we will compare the performance of the proposed system with its main competitors (at the time of the present article). The comparison metrics are based on (i) the cost of the system, (ii) the amount of balls that can be sanitized with one Euro, (iii) the time requested for one sanitization, (iv) the simplicity of use, (v) the certainty of the result.

The first type of alternative system is based on sanitizing spray gun dispensers, which an operator can use to distribute disinfectant solutions on the surfaces to be treated. This kind of systems are used to treat big spaces, and it is thought that they can be used also for sport balls. However, the antiviral effectiveness achieved by these systems is not certain, the amount of disinfectant solution sprayed and its distribution over the surface strongly depends on the ability and accuracy of the operator. In the case of sport balls, they need to be rotated at least once to reach the entire surface, and Personal Protective Equipment (PPE) gloves should be worn by the operator. Balls also need to be dried by means of a cloth or blotting paper after the treatment. While the cost of the system is low, the cost per treatment is still pretty high due to the cost of the disinfectant liquid. The cost of the spray gun is in the range of 100–200 €, while the cost per treatment depends on many parameters, considering a liquid cost of 8 € per liter to be dispersed in 10 min and 5 + 5 s per ball, we estimate a cost of 0.13 € per ball, or 7.5 balls per 1 €.

The second type of alternative solutions is based on automated liquid dispensing systems. We report on two possible implementations: A and B, both designed specifically for sport balls. The system A is a metal box with an external size of approximately 1 m^3^, it is equipped with an input hole where the ball is inserted, and a delivery system to collect the disinfected ball. System A uses 15 mL of a proprietary disinfectant liquid to sanitize the ball in 4 s. The ball is available in 6–8 s, but needs to be dry wiped: we expect the entire timing to be approximately 15 s. The retail price of apparatus A is approximately 950 €, a cost of 8 € per liter of liquid corresponds to a cost per ball of 0.12 €, or 8.3 sanitized balls per 1 € spent. The system B is also a box which can hold 8 sport balls in a 4 by 2 configuration, the balls are to be placed inside the box through an opening door and are disinfected with a proprietary liquid in 7 s. Similarly to system A, in this case, we expect 15 s for each ball including the time required to dry wipe the ball once the treatment is complete. The retail price of apparatus B is approximately 450 €, a refill has a cost of 55 € and guarantees 29 treatments (of up to 8 balls each), corresponding to a cost per ball of 0.24 €, or 4.2 sanitized balls per 1 € spent. In both A and B systems, the automated procedure should guarantee a certain disinfection result, even if no laboratory test are reported; however, the need to dry wipe the ball introduces a variable in the process since the towel might be also a source of contamination.

The system proposed in this paper does not require liquids, and has an important advantage in terms of simplicity of usage, which does not require wiping or contact with liquids. The required time of operation of 1 min per ball has been chosen to guarantee the treatment of tens of balls during the warm up time before the training, or the disinfection of the match ball during time-out (duration 1 min). If the timing is crucial, without modifying the optical structure, LEDs can be substituted with higher power devices; 280 nm LEDs of power up to 55 mW [23] are available and would allow a treatment time of approximately 10–15 s. The choice of 10 mW rated LEDs for the designed system is mainly a cost/performance optimization, a 10 mW LED retail price is in the range of 5–10 €, resulting in a price of the apparatus of approximately 1800 €. The cost advantage of the proposed system is related to its neglectable running cost: without any consumables required, the cost of disinfection is only related to electrical energy. Considering a wall plug power consumption of 50 W, the energy required for a disinfection cycle is just 0.83 Wh, corresponding to 5400 sanitized balls per 1 € spent (0.22 €/kWh is used as the average electrical energy price for the final user). To our knowledge, the systems based on liquids do not report specific antiviral results on the final sanitized object, but just report the effectiveness of the used liquid, thus not considering the effect of the surface material and the effective uniformity of the treatment. Summary of the comparison are reported in Table 3.

Concerning the usage of the proposed apparatus, we identified two possible scenarios: (i) an operator wearing PPE gloves positions the ball inside the chamber, which are then collected directly by the athlete at the end of the disinfection cycle, or (ii) one operator equipped with PPE gloves can position the ball inside the chamber using the left hand and collect the ball using the right hand (the chamber leaves enough room to lift the ball from the bottom) after the treatment is complete, and then deposit it on a sanitized surface. In this case, the lever should always be used with the same hand. The clear advantage is that the operation can be carried out by a single person, and since the ball does not get wet, it is possible to avoid contacts with other possibly contaminated surfaces when the athlete collects it.

**Figure 13:**
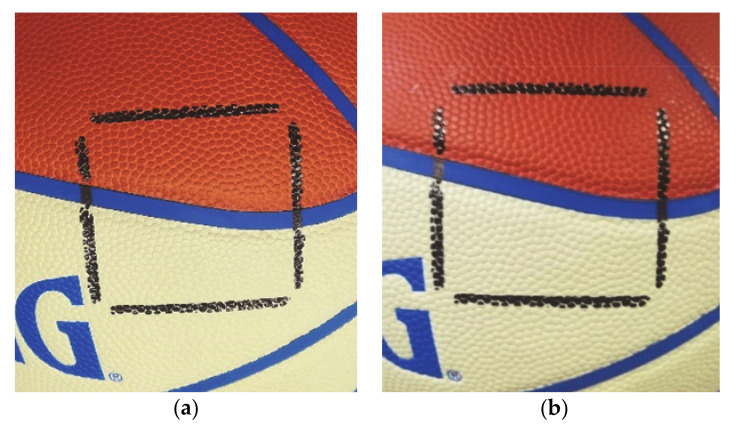
Results of degradation test on the surface of a regular basketball before (**a**) and after (**b**) 300 min of 275 nm radiation at an irradiance of 1.64 W/m^2^, amb. temperature is 25 °C.

When photons get absorbed, they might induce both heat and photochemical reactions on the irradiated material. Photons in the UV spectrum have higher energy with respect to visible ones, thus often causing discoloration and degradation of many organic polymers and natural materials [24] like plastic, rubber and leather: the typical materials of sport balls. It is therefore necessary to investigate on the possible effect of UVC radiation on the surface of the balls, and in particular in the degradation of their colors and mechanical properties. Degradation tests have been performed on a professional level basketball, where a square area of the ball has been subjected to a 275 nm radiation with the same irradiance of the prototype for an increasing time of up to 5 h and compared to an untreated area by means of visual and tactile comparison. Results are reported in Figure 13. After the test, no effects are visible or perceivable on the surface of the sample. To explain this behavior, we should consider the following: (i) UVC radiation is found to be less active on materials with respect to UVB radiation [25], (ii) the effects of UV radiation is enhanced by temperature, and in our case the short treatment time ensures a limited temperature increase (less than 5 °C); (iii) the irradiance has a low average value and a good uniformity on the surface of the target, thus not causing hot spots.

## 10. Conclusions

With this work, we presented an in-depth optical optimization analysis for the design of effective surface treatment systems based on 275 nm UVC LEDs, with a spherical volume of 18 L. The light sources are 10 mW LEDs emitting at 275 nm, which have been selected after an experimental comparison between different manufacturers and solutions. The design target has been the achievement of a minimum level of irradiance and optimization of the light uniformity; a spherical distribution of 56 LEDs has been chosen, together with a chrome plating treatment of the internal surface of the irradiation chamber.

A prototype of the designed solution has been manufactured, irradiance characterization reported a minimum irradiance of 1.385 W/m^2^.

The antiviral efficacy of the prototype has been demonstrated with respect to the SARS-CoV-2 virus by means of specific viral titering before and after the treatment: an inactivation greater than 99.9% has been detected after 1 min of exposure. The estimated dose for the treatment, calculated at the minimum irradiance, is 83.1 J/m^2^.

The designed system can treat any object which fits inside a sphere with a diameter of 250 mm, as long as its shape does not induce any shadows on other part of the structure (e.g., holes or pockets), irradiation times have been optimized for spherical targets and might require correction for objects with different shapes if the required fluence is not met on a specific area of the target.

The results of this study are of fundamental importance for the design of virus inactivation systems to be used in school and community objects, and can be readily implemented for the realization of UV-C-based systems.

## Figures and Tables

**Table 1:** LED selection and properties.

Manufacturer	LED Code	Nominal Current I_N_ (mA)	Nominal Flux @ I_N_ (mW)	Nominal Wavelength (nm)	Maximum Current (mA)	Forward Diode Voltage Vf (V)
A	A1	20	2–4	270–280	40	6.5
A	A2	350	37.5	275	800	6.5
A	A3	350	37.5	280	800	6.5
B	B1	150	7.5	278	150	7.5
C	C1	100	10	275	150	6.3
D	D1	50	5	278	100	6.1

**Table 2:** Results of the SARS-CoV-2 virucidal tests carried out on the surface of different sport balls.

Ball Type	Duration of the Treatment	Viral Titer Before Treatment *	Control Viral Titer on Same Surface *^,†^	Viral Titer after Treatment *	Percent Reduction after Test Contact Time
Soccer ball (3 repetitions)	1 min	7.0	7.0	0.01	>99.9%
	2 min				
Basketball (3 repetitions)	1 min	7.0	7.0	0.01	>99.9%
	2 min				
Volleyball (3 repetitions)	1 min	7.0	7.0	0.01	>99.9%
	2 min				

* Data express the log value of plaque-forming units per milliliter of viral suspension (PFU/mL) at different irradiation times. ^†^ For each ball, the control has been carried out on the same ball type and amount of time in dark condition.

**Table 3:** Comparison of the proposed system with other available systems.

Parameter	Manual Spray	Automated System A	Automated System B	Proposed System
Cost of the apparatus	~100–200 €	950 €	450 €	~1800 €
Sanitized balls with a cost of 1 € (only running costs)	7.5	8.3	4.2	~5400
Total time for treatment	~10 s	~15 s	~15 s	60 s
Simplicity of use	The operator needs to manually rotate the balls and calculate the correct timing	Automated timing	Automated timing	Automated timing
Certainty of the result	The result depends on the ability of the operator, only the liquid is tested	Only the liquid is laboratory tested	Only the liquid is laboratory tested	Laboratory tests conducted on the surface of the tested samples

## Data Availability

Data available on request due to restrictions related to commercial confidentiality agreements. The data presented in this study are available on request from the corresponding author. The data are not publicly available due to commercial confidentiality agreements with the manufacturer of the device.
